# Facilitators and barriers to engaging communities in health service research on dengue control in Indo-Pacific region: a systematic review

**DOI:** 10.1186/s12889-023-16845-8

**Published:** 2023-10-05

**Authors:** Cho Naing, Norah Htet Htet, Wong Siew Tung, Htar Htar Aung, Maxine A. Whittaker

**Affiliations:** 1 College of Public Health, Medical and Veterinary Sciences, James Cook University, Queensland, Australia; 2 School of Medicine, International Medical University, Kuala Lumpur, Malaysia

**Keywords:** Dengue, Health services, Facilitators, Barriers, Systamatic review

## Abstract

**Background:**

Dengue is a public health problem in the Indo-Pacific countries. There are concerns over the facilitators and barriers to community engagement in health service research aimed at dengue control. The objective of his study was to identify and synthesize facilitators and barriers to community engagement in health service research aimed at dengue control.

**Methodology:**

The Preferred Reporting Items for Systematic Review and Meta-analysis (PRISMA) checklist was used to perform this review. Health-related databases including PubMed, Ovid, and Google Scholar were searched for relevant studies. A consolidated framework with five domains was developed after undertaking a six-phase reflective thematic assessment of the data.

**Results:**

Thirteen studies were identified, spanning eight low-and middle-income countries of the Indo-Pacific region including Cambodia, India, Indonesia, Myanmar, Philippines, Sri Lanka, Thailand, and Vietnam. The studies in this review covered the period from 2002 to 2021. A broad range of study designs and objectives were revealed across these 13 studies. An array of communities such as the local government, project-related health staff, local health services staff, community leaders, local communities/residences/general public, heads of households, community health volunteers, school teachers, and schoolchildren participated in these dengue related studies. The five Consolidated Framework for Implementation Research (CFIR) domains of ‘intervention characteristics’, ‘inner setting’, ‘outer setting’,’ individual characteristics’, and ‘program implementations’ were used to identify and describe barriers and facilitators.

**Conclusions:**

The findings indicate a range of barriers and facilitators to community engagement in dengue control in the selected LMIC in the Indo-Pacific countries. Future health services research on dengue control approaches should be carefully planned, methodologically constructed, aligned with community engagement principles, and involve considerable community participation at all stages of the research.

**Supplementary Information:**

The online version contains supplementary material available at 10.1186/s12889-023-16845-8.

## Background

Dengue fever/dengue haemorrhagic fever (DF/DHF) is one of the neglected tropical diseases. This infection is caused by one of the four closely related dengue virus (DEN) serotypes (DEN-1, DEN-2, DEN-3 and DEN-4). As such, there is extensive cross-reactivity in serological tests, but infection with one serotype does not provide cross-protective immunity against the others. Hence, individuals living in an endemic area can be infected with each of the four dengue serotypes during their lifetime [1]. Approximately half of the world’s population is at risk of dengue fever/dengue infection, with 5.2 million recorded cases in 2019 alone [2]. According to the WHO, dengue is endemic in 129 nations, with the Americas, South-East Asia (SEA), and Western Pacific areas being the most seriously affected. According to the reports, Asia accounts for almost 70% of the global disease burden [2, 4]. To date, there is no specific treatment for dengue/severe dengue [2]. The first dengue vaccine was licenced in 2015 but its performance depends on serostatus [5]. One of the five technical elements of the WHO Global strategy for Dengue Prevention and Control 2021–2030 is engaged and mobilized communities [2, 6].

Many health services, particularly in low-and-middle-income countries, operate with limited resources. Community engagement is often required and used to complement the government services, increasing access to resources such as human, transport, labour as well as drawing upon local knowledge and experiences to enhance the effectiveness of the health programmes [7]. Community engagement is increasingly promoted in health services research (HSR), but the concept itself, and how it is best implemented in practice engagement, is understudied and contested [8]. HSR is defined as *“of scientific investigation that studies how social factors, financing systems, organizational structures and processes, health technologies, and personal behaviors affect access to health care, the quality and cost of health care, and ultimately our health and well-being. Its research domains are individuals, families, organizations, institutions, communities, and populations”* [9].

Community engagement in research stems from demands by community leaders, policymakers, and funders for meaningful community involvement to address health problems facing communities [10]. Numerous reviews have focused on community engagement in general health research [11–13], in which process or health outcomes were assessed.

Taken together, the purpose of the current study was to address the following question: What studies are available that identified facilitators and barriers to community engagement in HSR on dengue prevention and control? Hence, our objective was to identify and synthesize facilitators of and barriers to community engagement in HSR aimed at the prevention and control of dengue based on original research studies conducted in low-and middle-income countries (LMICs) of the Indo-Pacific region (table s1).

## Methods

This systematic review adhered to the Preferred Reporting Items for Systematic Review and Meta-analysis (PRISMA) (checklist s1). The current study was a part of larger study supported by TDR (the Special Programme for Research and Training in Tropical Diseases, cosponsored by UNICEF, UNDP, the World Bank and WHO) [Project ID AP21-00287]. A protocol is available from the authors on request.

### Study search

One investigator (NHH) searched studies in the health-related databases including PubMed, Ovid, Google Scholar, Cochrane Collaboration Library, Database of Abstract of Reviews of Effectiveness. The search was crossed checked by the second investigator (CN). The keywords with appropriate Boolean operators were used: “community engagement” “community participatory” “action research” “participatory research” “participatory action research” “community-based research” “dengue” “dengue fever " “dengue haemorrhagic’’ “dengue shock syndrome” “barriers” “enablers” “facilitators”. The search was extended to System for Information on Grey Literature including Social Science Research Network and Evidence for Policy Practice Information and Coordinating Community engagement (EPPI-Community engagement), Regional Bibliographic databases (e.g. Australian Education Index, AEI https://www.ace.org/my/library/Australian-education-index-aei). Search terms for Ovid are available in table s2. The search was limited to English language publications between 1990 and January 2023 in LMICs in the Indo-Pacific region (table s1).

### Eligible criteria

Individual studies were selected, if they.

1. conducted in the LMICs of the Indo-Pacific regions, regardless of study designs.
2. described community-based interventions or program in the primary health care settings on dengue/ severe dengue in which communities were engaged. Communities are as defined in the primary studies.
3. targeted the health service need within the areas of dengue fever/severe dengue.
4. reported at least one barriers or facilitators to community engagement as contextual factors.

Barriers and facilitators in this review were based on the definition as described elsewhere [11] with necessary modification for the focus of this review. Barrier is a factor that hinder progress towards community engagement. Facilitator is a factor that promote community engagement. Put simply, barriers and facilitators are factors that make community engagement more difficult or easier to achieve [14]. Community was defined as a group of people with diverse characteristics who are linked by social ties, share common perspectives, and engage in joint action in geographical locations or settings [15].

Studies were excluded, if they did not include participants from the specific LMIC in the Indo-Pacific region. Letters, case series, abstract/conferences proceedings without complete data, preclinical studies, experimental interventions with clinical outcomes or epidemiological studies that focus on the distribution of diseases were also excluded.

### Data collection

Two investigators (NHH, CN) independently selected the included studies using pretested data collection sheets. The two investigators independently collected the textual data relevant to our review question and objective. Collected information included study country, setting, type of intervention, study objectives, funding source, description of the HSR (structure, process, outcome assessment), description of the community, and facilitators/barriers encountered.

Any disagreements in these steps were settled by discussion with the third review author (MAW or WST).

### Data synthesis

Descriptive statistics for the important variables and reported as frequency/percentage (e.g., frequency of barriers) for categorical data, and mean (standard deviations, SD) for continuous data were undertaken. We planned to do meta-analysis if two or more studies provided numerical data of similar outcomes. However, due to a paucity of data in the selected studies, we were not able to undertake a meta-analysis.

### Assessment of the methodological quality

The methodological quality of the included studies was evaluated using the “Risk Of Bias In Non-randomised Studies-of Interventions” (ROBINS-I) tool [16]. The ROBINS-I tool captured seven domains of bias (i.e., confounding, the selection of participants, measurement/classification of interventions/exposures, deviations from intended interventions/exposures, missing data, the measurement of the outcomes, and selection of the reported results). Two investigators (WST, CN) independently assessed the methodological quality, and any discrepancies were settled by discussion.

### Identification of barriers and facilitators to community engagement in HSR

To synthesise results across studies, we adopted standardised terminology for terms such as leader, stakeholder and staff, adapted from the Cooper [17] with necessary modifications for the focus of the review, namely community engagement. In the current review, leaders are those in management or leadership positions or senior positions responsible for coordination of the community engagement activities for DHF control. Staff refers to all staff within an organisation across the different levels of hierarchy, and stakeholders refer to any individuals or partner organisations with a role in the intervention.

Synthesis of data involved the two steps such as thematic analysis and mapping of thematic synthesis into a determinant framework (i.e., Consolidated Framework for Implementation Research (CFIR)).

### Thematic analysis

First, we undertook the six phases of reflexive thematic analysis (RTA): (1) familiarising oneself with data; (2) generating codes; (3) constructing (initial) themes; (4) reviewing potential themes; (5) defining and naming themes; (6) producing the report [13]. NHH started following familiarisation of the relevant data through screening, selection, and then multiple readings of the studies identified during the stage of study selection. The two investigators (CN, NHH) developed consensus on data coding practices in phase 2. NHH generated initial themes in phase 3, and initial themes were reviewed and developed during phase 4 by CN, and were consulted with MAW, who has extensive experiences in qualitative studies. Additionally, two other investigators, WST and HHA, gave feedback on the grouping and naming of themes in phase 5, and finally produced a report in phase 6. Following each phase of RTA reinforced the need for investigators to have deep immersion into the data as well continuous reflexive accounts [18].

Secondly, the report extracted from thematic analysis was mapped into the five domains of CFIR framework. Details of the CFIR framework are available elsewhere [19].

## Results

### Search outcome

**Fig. 1:**
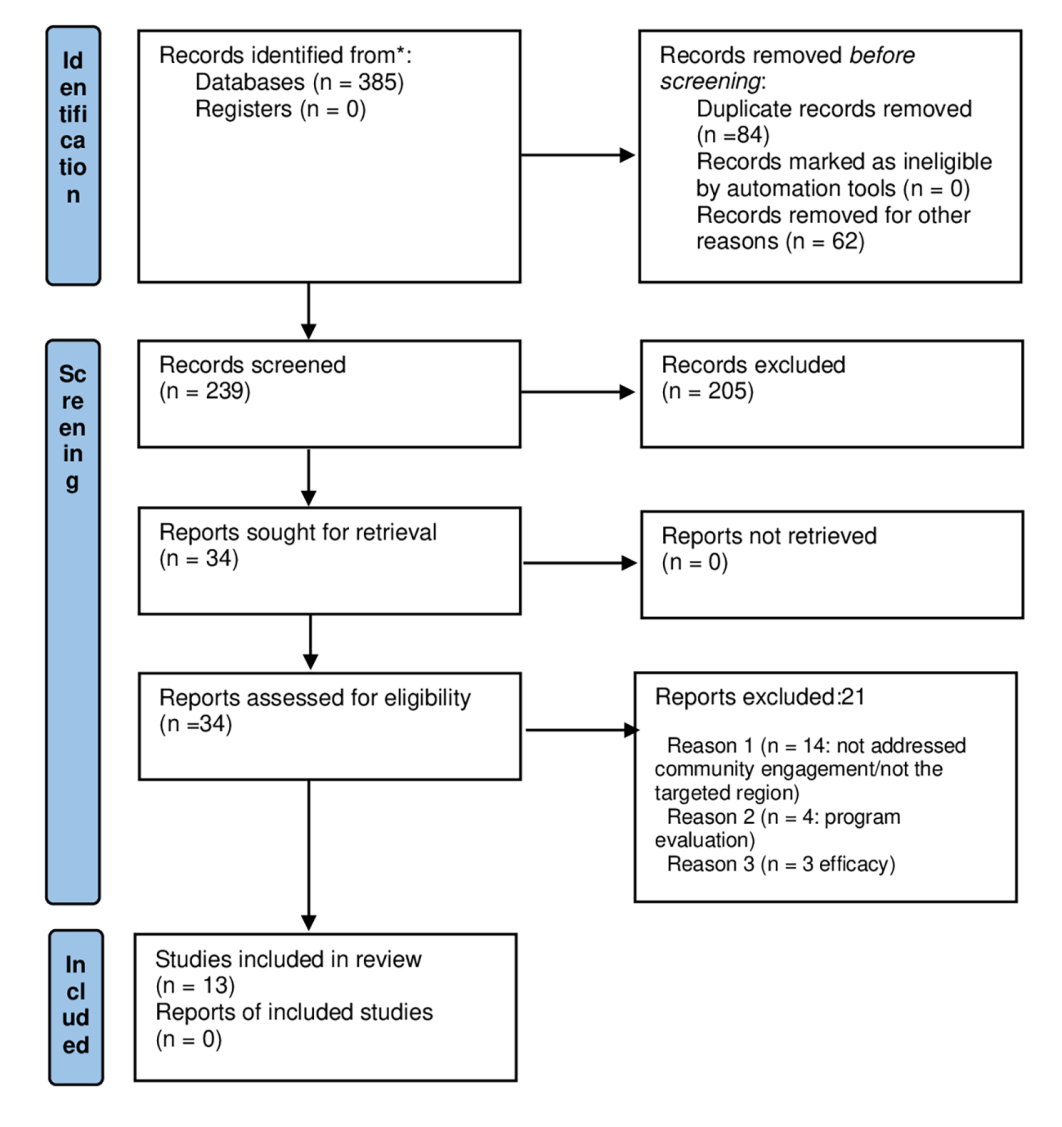
Study selection process

Figure 1 presents the study selection process. Of the 385 records identified, 79 duplicates were removed, and 274 titles and abstracts were screened. Of these, 34 studies that were deemed relevant were assessed in full-text and a final of 13 studies [20–32] met the inclusion criteria. The reasons for exclusion of 21 studies are provided in Table s3.

### Characteristics of the included studies

Table 1 provides the characteristics of the included studies. They were conducted across eight countries in the Indo-Pacific region (i.e., Cambodia, India, Indonesia, Myanmar, Philippines, Sri Lanka, Thailand, Vietnam). Of these eight countries, the majority are lower-middle income countries (6/8, 75%) such as Cambodia, India, Myanmar, Philippines, Sri Lanka, and Vietnam. Of 13 studies identified, the most frequent studies were conducted in Thailand [26, 29, 31] or Vietnam [24, 25, 30]. Three studies (3/13, 23%) were published in 2012 [23, 30, 32], while the earliest and latest studies in this review appeared in 2002 [24] and 2021 [31].

**Table 1:** Characteristics of the studies identified for this review

No	Study author & year [ref no.]	Country	Study objective	Design	Approach	Types of community
1	Abeyewickreme 2013 [20]	Sri Lanka	to assess the validity of assumption that waste management through community mobilization can reduce breeding places at HH level	prospective interventional study	FGD & IDI	community volunteers, community & project staff. All senior students (Grade ≥ 10), teachers & school principals
2	Asri 2017 [21]	Indonesia	to describe the existing social capital in a community & how it was used to fight DHF.	qualitative descriptive	IDI	social groups, strong leaders, community volunteers
3	Echaubard, 2020 [22]	Cambodia	To describe the processes of community engagement & transdisciplinary collaboration underpinning community-based dengue management in rural primary schools & HHs.	case study based on a qualitative data collection.	community dialogues, participant observations, FGD, IDI	CHW, health centre chiefs, school directors, monks, priests, teachers, farmers & members of the local education office, school children & their parents
4	Espino 2012 [23]	Philippines	to examine responses to introducing water container management to control dengue vectors in two diverse communities in Masagana City.	community-based intervention study	FGD & IDI	HH members, local government & health officials, barangay captains & homeowners’ association presidents; city sanitation inspectors, CHWs or BHWs
5	Kay 2002 [24]	Vietnam	to determine whether the communities viewed dengue and DHF as a serious health threat; to determine their knowledge of the etiology, attitudes, and practices regarding control methods including mesocyclops; and to determine their receptivity to various information methods.	Community-based research	community-based methods on surveillance	CHV, trained health workers, residents, schoolchildren. teachers, of schoolchildren.
6	Kay 2010 [25]	Vietnam	to evaluate whether or not the programs were still being maintained 7 years and 4.5 years after formal project activities had ceased, respectively.	CS descriptive study	FGD & IDI	health collaborators, SC,& community members in the study communes. 26 key informants, project managers at the national level, community POs, POS from the provincial centers of preventive medicine, POs from DHC district health centers, representatives from two different communal people’s committees, health staff from different communal health centers, & heads of different primary/secondary schools.
7	Kittayapong 2012 [26]	Thailand	To report the successful application of an eco-bio-social or ecohealth approach to dengue prevention and control in urban and peri-urban settings in eastern Thailand.	CPAR	cluster RCT approach	community leaders, local administrative authorities, municipal mayors, local public health officers, & communities.
8	Lwin 2017 [27]	Sri Lanka	To combat the issues of an exhausted dengue management system and to make use of new technology, in 2015, a mobile participatory system for dengue surveillance called Mo-Buzz was developed and launched in Colombo, Sri Lanka.	CPAR	digitalized surveillance	PHIs, general public
9	Mathur 2020 [28]	India	to ascertain the existing KAP n dengue hotspot areas and empower them for vector borne disease control using a HE models and also to find different challenges and barriers faced by frontline health workers during vector borne disease surveillance.	CS followed by intervention	IDI	ASHAs, 4 ANMs, 1 LHV, 2 MOs in Pratapnagar, HH members
10	Murray 2014 [29]	Thailand	to test the effectiveness of permethrin-impregnated school uniforms for dengue prevention in Chachoengsao Province	community acceptability survey mixed-method approach including double-blind, cross-over RCT	FGD & IDI	investigators (health staff), teachers, parents, students
11	Nguyen-Tien 2019 [30]	Vietnam	to explore the barriers to the implementation of CE in a dengue VC program in an urban district of the Hanoi city.	qualitative study	FGD & IDI	community members, stakeholders,
12	Suwanbamrung 2021 [31]	Thailand	to develop risk dengue village prediction criteria, predict village dengue risk, and strengthen dengue prevention based on community participation	CPAR	5 phases approach: (i) preparing the community, (ii) developing assessment criteria for assessing dengue risk village, (iii) application of a computer program (http://surat.denguelim.com), (iv) predicting dengue risk villages, (v) utilising the findings for village dengue prevention	stakeholders, who involved dengue prevention of village, LAO, PHP, community leaders in villages, VHV, heads of hospital, HHs & community
13	Wai 2012 [32]	Myanmar	To build up and analyse the feasibility, process, and effectiveness of a partnership-driven ecosystem management intervention in reducing dengue vector breeding and constructing sustainable partnerships among multiple stakeholders	community-based intervention study	FGD & IDI	Multi-stakeholder partner groups (Thingaha) & ward-based volunteers

AGO: Autocidal gravid ovitraps; ANM: Auxiliary Nurse Midwife ASHA: Accredited Social Health Activists CBOR: Community Based Operations Research; CE: community engagement; CHV: communal health volunteers; CHW: community health workers; COR: Community Operational Research; CPAR: Community participatory action research; CS: Cross-sectional study/survey; DHC: district health centers; DHF: dengue hemorrhagic fever; EWARS: Early warning and adaptive response system; FGD: Focus group discussion; HE: health education; HH: household; IDI: in-depth interview; IVM: Integrated vector management; KAP: knowledge, attitudes and practices; LAO: local administrative organization; LHV: lady health visitor; MO: medical officer; PHI: public health inspectors; PHP: public health provider; PO: project officer; RCT: randomised controlled trial; SC: school children; VC: Vector control; VFT: Value Focused Thinking; VHW: village health volunteer

Several research designs such as three cross-sectional surveys [25, 28, 29], community-based interventions [23, 24, 32], or community-participatory (action) research [26, 27, 31], three qualitative designs [21, 22, 31], and one prospective experimental study [20] were implemented. A broad range of objectives emerged across these 13 studies in eight countries (Table 1). However, none addressed barriers/facilitators as the primary aim of the study, instead addressing these as part of the findings, or limitations.

### Types of participating communities

A broad range of communities participated in these dengue-related studies, including local government, project-related health staff [23, 26], local health services staff including auxiliary nurse midwives (ANWS), accredited social health activists (ASHAs) [28], community leaders [22, 26], local communities/residences/general public and head of households [24, 27, 30], community health volunteers [22–24], school teachers and school children [22, 24], faith leaders [22], and multi-stakeholder partner groups [32]. Their participation was predominantly described as being participants involved in focus group discussions (FGDs) and/or in-depth interviews (IDIs).

### Methodological quality

**Fig. 2:**
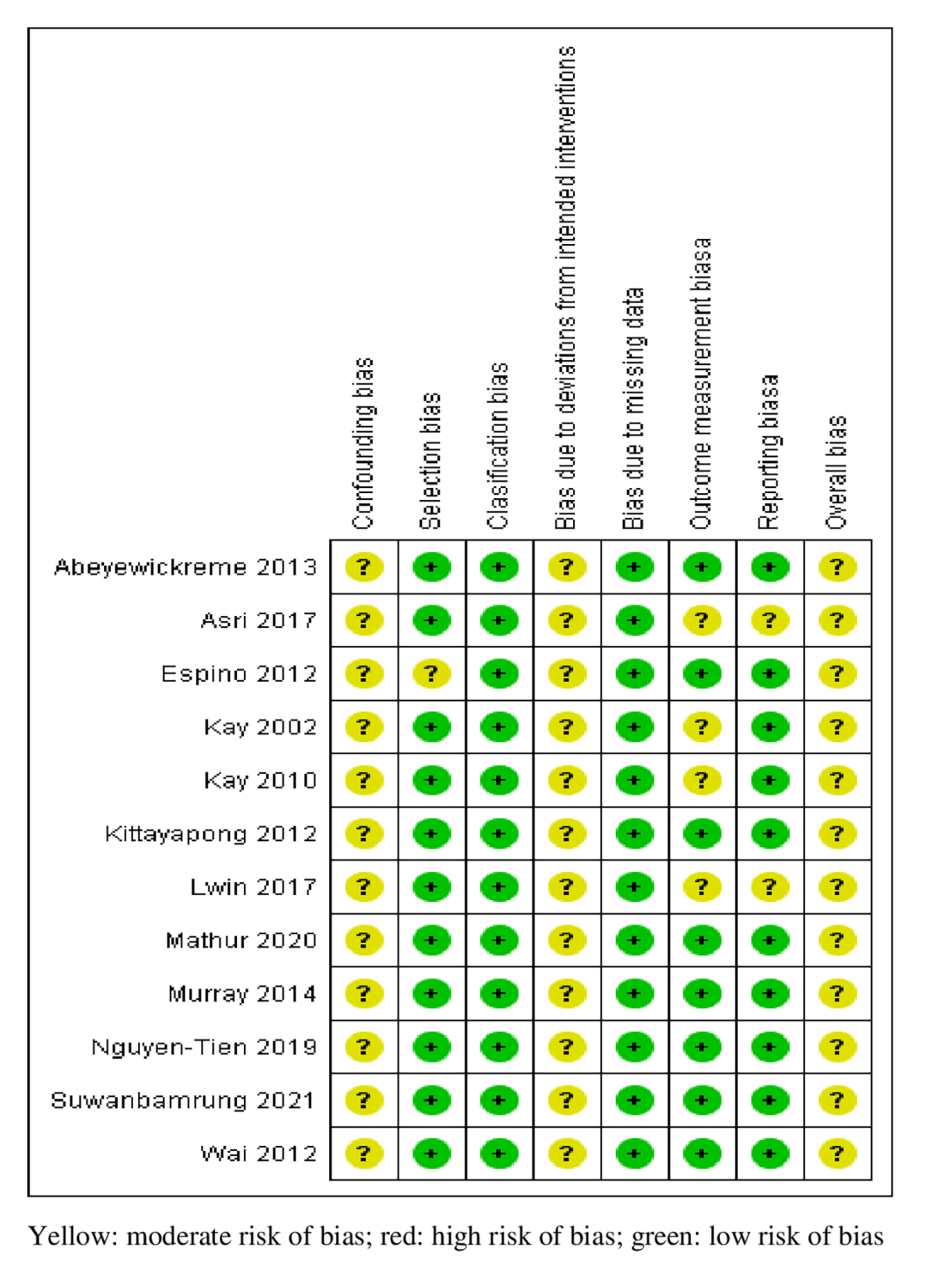
Quality assessment of the studies

In terms of methodological quality, the studies were assessed to be at either moderate or high risk of bias in domain-based assessment; none of these studies were rated to be at low risk of bias. Overall, the quality of the studies was at moderate risk of bias with all 13 studies mainly due to the concerns over confounding bias and bias related to deviations from intended intervention (Fig. 2).

### Barriers and facilitators

The organizing and grouping of the barriers and facilitators were identified using the five CFIR domains, namely intervention characteristics, inner setting, outset setting, individual characteristics and “programme implementation” (Table 2). In many instances, the opposite side of a problem, challenge, tension, or barrier was framed as a facilitating factor or ‘enabling factor’. For instance, having a variety of stakeholders involved allowed for the facilitation of a broad range of perspectives on the research topic. On the other hand, having low involvement of stakeholders involved in the pre-implementation requirements was viewed as an impediment.

**Table 2:** Summary of findings under CFIR Constructs

Domains	Barriers	Facilitators
**Intervention characteristics**		
■ Evidence strength and quality		
■ Relative advantage	N/A	■ Local manufacturers were explained to produce tightly fitted lid covers at low cost (p 463) [32].
■ Adaptability	■ Vertical interventions in dengue vector control are not sustainable particularly if they are built upon ‘crisis management’ as in our case the response to the dengue threat after cyclone Nargis (p 467) [32]. ■ Most of the general public was unwilling to participate in the survey [27]. ■ The participants felt that it would be the government’s responsibility to ensure good dengue control [29].	■ The extent of interest of the community, acceptance, and their active participation including the development of a sense of the responsibility (p 467) [32]. ■ Factory-impregnation of the school uniforms could not be detected via smell or other changes in the textiles [29].
■ Complexity	■ *“This work belongs to health station. So, it’s very hard for us”* (p 967) [30].	
■ Design quality and packaging	■ Most were not mobile technology literate [27].	*“Co-design of dengue prevention communication material by students and community members”* (p.8) [22] Specific groups of “ecohealth volunteers” were established in each community, who were then engaged in regular dialogue with local community leaders and coordinators [32].
■ Cost	■ Budget is usually low and perceived as inadequate to undertake the duties required (p 969) [30]. ■ *“The participants work hard but the allowance is too little, therefore, nobody certainly feels happy”* (p 969) [30].	■ *“If the budget increases, the interaction between WPC and the stakeholders will be better*” (p 969) [30]. ■ Sustainability of the current intervention package depends upon………. additional programme costs (p 467) [32].
**Inner setting**		
■ Structural characteristics	■ Dynamic disease mapping component (i.e., hotspot mapping), although useful for them, was not deemed necessary for the public. They were worried that it might trigger outbreaks of panic [27]. ■ Distribution of responsibility on disease control between two important sides of WPC and WHS was not balanced (p 966) [30].	N/A
■ Networks and communications	■ Limited stakeholder participation [31]. ■ Poor attendance at the project inception negatively affected engagement in the project [23]. ■ Communication with stakeholders was problematic, as the Mo-Buzz research team was based in Singapore (i.e., language problem) [27]. ■ Knowledge gaps between the provider and communities: *“Even there are many participants in PEM but only a few people expressed their idea in the meeting”* (p.9) [22] ■ “….*because some children can learn but they cannot explain to their parents*….” (p.8) [22].	■ Multi-partnership approach: the EFG acted as the liaison between the community and the THD. They established close relationships among different partners and helped to build and maintain the sense of ownership [27]. ■ Careful choice of local coordinating committee members and of the health volunteers [24]. ■ trained ecohealth volunteers informed householders of general knowledge regarding dengue prevention measures, the public health services and local administration in collaboration with the university research teams provided materials and resources [26]. ■ Bench conferences in village setting [23]. ■ High level stakeholder meetings with official representatives of government bodies & community leaders to prepare the planning/ monitoring/evaluation of intervention activities, & to help to mobilize local resources and give logistical support (p.9) [22]. ■ The students communicated their material to an audience of 20–45 villagers in each session with the support of CHW for the design of specific messages (p.9) [22].
■ Culture	■ Cultural barriers persisted in the management of spiritual bowls; the use of dragon fly nymphs for cement tanks still needs promotion (p 466) [32]. ■ Using serial blood samples from an apparently healthy population was the culturally based resistance to the collection of blood (p 47) [24].	N/A
■ Learning climate	N/A	■ Each group comprised one leader and four core members. They were trained for information dissemination and how to manage vector control tools (p 463) [32]. ■ Communication problem was overcome by learning about Sri Lankan customs and culture from Sri Lankans living in Singapore and by undertaking field trips and interviews [27].
■ Leadership engagement	■ “Mass organizations and community leaders are lacking in enthusiasm”, “*Frankly speaking, the role of mass organizations, for example women’s union, youth union in some wards is still only doing for show as a movement” (*p 967) [30].	■ Distributing pamphlets and booklets and assisting people in the application of targeted container interventions strengthened the leadership of EFG and the development of sense of ownership by community members (p 464) [32]. ■ Head of CHC, vice head of central project commune, and head of school provided leadership for dengue control [25]. ■. Since Risma became mayor, village leaders were asked to focus on dengue fever, because it was a health concern not only for the Department of Health. Risma said this was your region; if people are sick, you have to help mobilize society (p 376) [21]. ■ Monks, who received HE on dengue control, have proposed ceremonial occasions at the pagoda as acceptable moments to communicate dengue related control knowledge or procedure [22].
■ Available resources/Staff/	■ There was fewer staff to service large catchment areas [28]: “*We need more staff for surveillance as area is big and we have 10 ANM’s. We have been allotted 5 wards and population is 1 lakh. To cater such a big population, ANM’s should be double in number”* (p 1759*)*. ■ Inadequate manpower and vehicles for ward-based waste collections [32]. ■ Overburdened workloads [30].	A BHW said that household member in a village in Philippine told her, ‘*It is good that we are being visited by you and that there is a project like this’* [23].
■ Readiness	■ Lack of readiness and activeness (p 968) [32].	N/A
■ Access to knowledge and information	■ Lack of knowledge and preparedness also has been the result of ineffective community engagement (p 967) [30]. ■ Communities (Haiphong residents) treated information from local officers with greater skepticism [24].	■ information from the trained health workers in the team was respected because rural commune residents develop a closer relationship with their elected representatives than with city dwellers [24].
■ Organizational incentives and rewards	■ *“I think the caring with community leaders on allowance and spiritual issues is lacking, so they were unenthusiastic”* (p 967) [30].	■ A compactor was financed through a small grant scheme to improve efficiency, and a percentage of the profits was returned to the commune to facilitate the payment of health volunteers after the project ceased [24]. ■ incentives as a facilitating factor for success in reducing of dengue incidence. “*Because they received it (pocket money), changes occurred. ….* ” [21]. ■ *“This monthly allowance was sufficient to encourage collaborators to continue implementing their tasks*” (p 829) [25].
**Outer setting**		
■ Cosmopolitanisms	■ WPC should take the lead and organize the dengue vector control activities under the consultation of WHS. However, the WPC handed over almost all work related to health protection to the WHS (p 966) [30].	N/A
■ External policy and incentives	■ Launching of the system was delayed as a political election was underway and had implications for government approvals [27]. ■ Lacking local government leadership (p 459) [23]. ■ Attrition of EFG members and volunteers required the need of the development of a system for volunteer replacement [32]. ■ When a new chairman of WPC takes up the duty, everything has changed. He said that there is no need to do this work (p 967) [30].	■ Sustainability of the current intervention package depends upon political commitment and continuing support by the local governance [32]. ■ Leadership of EFGs was successful as the ward authorities developed a strong commitment in problem identification (p 467) [32]. ■ Incentivize reports submitted by the PHIs [27].
**Individual characteristics**		
■ Knowledge and beliefs about the intervention	■ Because dengue control was their (city health officers) responsibility, the honorarium task force members had stopped (p 459) [23].	N/A
■ Self-efficacy	■ Language posed a barrier. The main mode of communication in Sri Lanka is Sinhalese or Tamil, but none of the researchers were proficient in either language [27]. ■ All physicians in the district hospital should be trained (for using the DPCG) (p 100,168) [31]. ■ Health workers need to have qualification and being trained to improve the persuading skills while working with community (p 967) [30].	■ An external translator who was fluent in Sinhalese, Tamil, and English was employed to overcome the language barrier [27].
■ Individual identification with organization	■ Stakeholder communication was made more difficult by differences in opinions borne of views that were influenced by different specialties [27].	■ The leadership of EFGs was successful as they achieved that ward authorities developed a strong commitment in problem identification at baseline and in scheduling, motivating people, and distributing intervention materials and later on in monitoring the implementation and results (p 467) [32].
Individual stage of change	■ They (communities) also have not cooperated and followed the health staff’s instructions for dengue vector control (p. 968) [30]. ■ A sense of fear of the ANWS and ASHAs in engaging communities [28]; *“One incident occurred with me when I was alone during the survey and I visited one house. That man ran after me to hit me with his stick. So, there is always a sense of fear in mind”* (p. 1757).	N/A
**Process Implementation**		
■ Planning	■ Implementing rules and/or regulations were absent (p 459) [23].	■ Six multi-stakeholder groups involved, and in the first phase, each stakeholder group were thoroughly discussed about power, legitimacy, interests, and interactions towards controlling dengue vectors [32].
■ Key stakeholders	■ The DPCG needs all stakeholders to participate, integrate, and coordinate for continued monitoring and use (the guideline) (p 13) [31]. ■ Involving multiple stakeholders was a challenge to ensure the sustainability of the intervention [20].	The cross-sectorial collaboration & transdisciplinary action that took place during the school-based sessions together with the strong engagement of students in activities of knowledge sharing in communities, led the department of school health of the MoE to incorporate the co-designed dengue curriculum into the national school program [22].
■ Reflecting and evaluating	■ Lack of interest and an attitude of dependency on action from the health sector of local people’s committee [30]. ■ *“The common problems that people complained were less loudspeakers and unclear sound… The communication by loudspeakers is bad*” (p 969) [32]. ■ The participants felt that it would be the government’s responsibility to ensure good dengue control [23]. ■ *“Encouraging the community itself to change is rather difficult*.” (p 375) [21].	■ Favourable and unfavourable conditions related to the six strategic options to reduce dengue vector breeding was discussed (p 465) [32]. ■ Reflection of meeting discussion [31]. ■ community members indicated that guppies (i.e. vector control tools) were informally distributed outside interventions areas, suggesting knowledge transfer, cultural acceptability, strong feasibility of scaling up & project outcome sustainability [22].
■ Champions	■ Because low attendance to the training session. The issue is who is a suitable champion for strategies in dengue control (p 459) [23]. ■ A form of social capital, which plays an important role in the efforts to eradicate dengue fever is Sanitarian. They were responsible for the entire DHF prevention and control program in the region and implemented it in their own PHCs. They have to cooperate with the village offices, the sub-district office, the regional DOH, volunteer larvae observers (Bumantik, Jumantik, Rumantik, and Wamantik) in each region [21].	■ Sending one of the more technically proficient PHIs to Singapore to learn the system well. The PHI then tasked with troubleshooting issues faced by other PHIs. The PHI returned to Colombo as an **ambassador** to train his fellow PHIs and help promote the usage and troubleshoot. Following this training, uptake of the app increased [27].

ANW: aauxiliary nurse midwives; ASHA: Accredited Social Health Activist; BHW: Barangay health workers/Community health workers; Bumantik (women larvae observers); CFIR: Consolidated Framework for Implementation Research; DOH: Department of Health; DPCG: dengue patient care guideline; EFG: Eco-health friendly groups; Jumantik: Family larvae observers); MoE: Ministry of Education; MoH; Ministry of Health; PHIs: Public Health Inspectors; Rumantik : Teacher larvae observers; THD: Township health department; Wamantik: Women student larvae observers; WPC: Ward people committee; WHS: Ward health station

The following are outlines of the five CFIR domains, which were attributed to the barriers/facilitators mentioned in the studies selected for the current review. The respective constructs are presented in Table 2.

### Intervention characteristics

The health issues that were the focus of the intervention to be accepted, adapted, tailored, refined, or reinvented to meet local needs was reported as barriers to community engagement in four studies (4/13, 31%) [27, 29, 30, 32]. For instance, the actual vertical nature of the health programme was a barrier to engagement, whereas having a more integrated service facilitated engagement [27, 32]. A low budget that was inadequate to undertake the duties required [30] or the perception that dengue control was solely a government responsibility [29] were identified as barriers.

Three studies (3/13, 23%) reported enabling factors [29, 30, 32]. The availability of the required materials from the local manufacturers [32], quality of materials [29], having an adequate budget, and having communities’ supporting any additional local costs [30] were identified as facilitating factors for community engagement in interventions. Moreover, the involvement of trained specific groups of ‘eco-health volunteers’, who were then engaged in regular dialogue with local community leaders and coordinators for mobilizing dengue vector control activities in their communities [32] was a facilitator.

### Inner setting

Seven studies (7/13, 53%) identified challenges such as a lack of enthusiasm of the participating stakeholders, insufficient knowledge about the intervention, lack of incentives, inadequate networking and communications, concern about the extra workload, a shortage of human resources, and cultural barriers [22–24, 27, 29, 31, 32].

For instance, the extra workload was discussed as a barrier because the extra number of people participating created a larger population to serve by an already stretched number of staff [27, 29]. Another study described the issue of inadequate availability of human resources and vehicles for ward-based waste collections [32]. Limited stakeholder participation [31], and poor attendance at the project inception [23] were described as barrier to engagement in the project. Another study highlighted that cultural barrier such as using serial blood samples from an apparently healthy population were the culturally based resistance to the collection of blood [24].

Numerous factors that promoted community engagement were identified in nine studies, including the use of existing community networks and active communications between the researchers and the communities, the commitments of local leaders, the involvement of trained volunteers, faith leaders, and local schools to the intervention, and the availability of funding [21–27, 32]. For instance, ‘bench conferences in village setting as a means of actively communicating with the community [23], high-level stakeholder meetings [22], receiving pocket money as incentives [21, 24, 25], the commitments of the head of local authority such as mayor (Risma) [21], and the involvement of monks could transfer knowledge on dengue during ceremonial occasions [22] were enabling to community engagements.

### Outer setting

Four studies (4/13, 31%) addressed various challenge such as the lack of local government commitments, volunteer attritions, frequent changes of local leaders, and heir accountability n under this domain [23, 27, 30, 32]. For instance, there were delays in launching he intervention due to political elections [27], a lack of local government leadership [23], frequent changes of the chairperson, and a lack of accountability of the ward’s People’s Committee [30], and attrition of volunteers and inadequate system for volunteers’ replacement [32] were also barriers to community engagements.

Within the outer setting, having sustained support and commitments and offering incentives for reporting were facilitating factors addressed in two studies [27, 32].

### Individualised characteristics

An array of challenges including a lack of interest or enthusiasm within the target community, or lack of suitable skills/qualifications amongst the health staff providing the intervention, dependency of individuals, and community members having a sense of fear of the health providers/volunteers of engaging with communities were barriers to community engagement in four studies [23, 27, 28, 30]. For instance, when the community perceived hat dengue control was the city health officers’ responsibility [23] they were less engaged. The lack of suitable skills or experience included the language barrier [27], and the need for health workers’ knowledge and skills in persuasion and motivation, while working with the community [30] were described as barriers. A sense of fear of the ANWS and ASHAs in engaging communities was also reported [28].

On the positive side, the unique role of ward (community) leaders and their commitments, strong coordination with other stakeholders, and effective communication in local language proficiency seemed to be facilitating factors described in two studies [27, 32].

### Processes of implementation

Six studies (6/13, 46%) reported numerous challenges such as lack of rules/regulations supporting engagement processes, lack of champions, difficulties arising due to frequent substitution of stakeholder’s representatives leading to lack of continuity and commitment, low involvement by the communities in pre-implementation activities as barriers to community engagement [20–23, 30, 31]. For instance, it was difficult to identify a suitable champion for strategies in dengue control due to low attendance numbers in the provided training [23]. The involvement of multiple stakeholders created a challenge in ensuring the sustainability of engagement in the intervention without the input of the research team [21]. Also, a lack of implementing rules and/or regulations [23], and difficulties encountered in encouraging the community to change [21] were barriers to community engagement in implementation.

Four studies (4/13, 31%) described the key facilitators under the domain of ‘process implementation’ [22, 27, 31, 32]. For instance, engaging stakeholders in pre-implementation activities or discussions during the process made empowered them to be self-reflective, which in turn assisted in conflict resolution and a better understanding of the objectives of the work and more competency in team work [31, 32] or codesign of intervention [22]. Also, recruiting and identifying the champions as the ambassadors of teamwork [27] was of paramount importance to operate in the community’s capacity.

## Discussion

This review included 13 studies that fulfilled the criteria for inclusion, most of which came from lower-middle income nations in the Indo-Pacific region and contextualized a number of barriers and facilitators to implementing community engagement in HSR aimed at dengue control. Key findings were identified under the five CFIR domains, namely intervention characteristics, inner setting, outset setting, individual characteristics, and programme implementation. To our knowledge, this is the first review that has addressed barriers and facilitators to community engagement in HSR aimed at dengue control that used the CFIR domain in reporting. Studies included in this review were from the high burden Asian countries, as reported by WHO [2], reflecting a certain degree of geographic representativeness.

A published systematic review of the use of the CFIR domains reported that a comprehensive understanding of barriers and facilitators might serve as a means to improve the implementation of the intervention. However, there was a lack of international synthesis on this aspect [33]. The current review attempts to fill such gap in community engagement in HSR aimed at dengue control in the selected countries.

A key message derived from the current analysis is that there are challenges in conducting effective community engagement across all stages of the HSR process. There are concerns about where the priorities should lie. According to a published systematic review that addressed the benefits of community-based research, the partnership between researchers and communities fostered a process of co-learning and empowerment and made it easier for them to share knowledge, skills, capacity, and power with each other [35]. However, as mentioned in this review, the opportunity for co-learning was limited by the stakeholders’ lack of suitable participants. Some but not all studies included in this review addressed the challenges and opportunities linked to the five CFIR domains and underlined the value and implications of partnership synergy across various stakeholders.

The level of effectiveness of partner communication and the extent of stakeholder participation were the two distinct CFIR constructs noted as facilitators, and in the converse, obstacles that were most commonly described in this systematic review. Similar to a published systematic review of polio vaccination programs [37], the current review encountered three major obstacles: a lack of enthusiasm by stakeholders, a lack of incentives for community participation especially for volunteers, and insufficient staff in the government programs delivering the intervention. Therefore, as indicated earlier [14, 37], considering creative ways that motivate and encourage staff to visit rural areas and vulnerable groups may be the core for effective implementation. Additionally, this review identifies networking and communication as essential elements for community engagement. A published study suggested [37] that this kind of communication (via networking or local gatherings) would support the development of a new and deeper understanding of the issues by the stakeholders, supporting them to reflect on actions undertaken and information obtained and then to inform them about further action. To facilitate communication, language and cultural obstacles were identified as needing to be addressed, reflecting the necessity to focus on these, for example, by using a translator in all communications prior to implementation, in order to facilitate building and sustaining the desired partnerships.

Building capacity to improve health involves the development of sustainable skills, resources, and organizational structures in the affected and vulnerable communities [12]. A study on the assessment of community engagement in Partnered Mental Health Services Projects [36] reported that community engagement in research and partnership size impact both partnership functioning and outcomes. A well-functioning partnership will support synergy among the members of the partnership and affect outcomes.

### Public Health implications

The findings identify studies that addressed a variety of barriers and facilitators to community engagement in dengue control of the LMICs in Indo-Pacific region. When considering ways to address the identified barriers as observed in our analysis, it was more valuable to focus on the use of scarce time and resources of the trained community members and health practitioners to involve them in interpreting and making sense of the data, as highlighted by Israel and associates [34, 35].

Research projects involving communities, such as dengue control in our study, require academic members to become part of the community and community members to become a part of the research team. This would have created a distinctive working and learning environment before, during, and after the research [11]. However, a published study has highlighted that **w**hile partnered research projects (a term used interchangeably with community engagement) have the potential to address pressing community health issues, there is little empirical evidence about the impact of the degree of community engagement in research on outcomes of the projects [36]. The studies identified for the present review did not explicitly discuss outcomes from community engagement, thus not addressing this gap.

Among the factors that motivate people to participate in health (and HSR) are wanting to play an active role in bettering their own lives, fulfilling social or religious obligations, feeling a need for a sense of community, and wanting cash or in-kind rewards [12]. Although not all factors were explicitly identified in the studies included in the review, some did report how the lack of cash incentives was a barrier.

### Study limitations

We acknowledge some limitations. First, the search did not include studies that were published in languages other than English. Useful evidence in published or grey literature written in languages other than English could be overlooked because of information bias. Second, no information on gender-based community engagement was provided in the studies that we included in this review. As a consequence, there is a shortage of gender equity evidence in this review. Thirdly, due to a multi-context approach in data synthesis, the findings might be too general, overlook specific contexts, and blur the critical differences between the included studies.

Nevertheless, the current review has some advantages. This systematic review addressed the systematic collection of community input and experiences in dengue research. Studies of the eight endemic countries where dengue is a high burden were identified. The findings highlighted that community-based research recognizes social structures and social processes that support or develop community members’ capacity to collaborate to improve health [37]. By utilizing a CFIR determinant framework, the findings contribute to the limited body of knowledge from an implementation perspective. The factors identified in the present review may assist researchers, policymakers, volunteers, and community members in developing better plans and designs for community engagement in health service research on dengue control programs.

## Conclusions

The findings indicate a range of barriers and facilitators to community engagement in dengue control in the selected LMIC of the Indo-Pacific countries. Future health service research on dengue control approaches should be carefully planned, methodologically constructed, created in accordance with community engagement principles, and involve considerable community participation at all stages of the research.

### Electronic supplementary material

Below is the link to the electronic supplementary material.

Supplementary Material 1

Supplementary Material 2

Supplementary Material 3

Supplementary Material 4

## Data Availability

All data generated or analysed during this study are included in this published article and its supplementary information files.

## Abbreviations

ANWS: Auxiliary nurse midwives
ASHAs: Accredited social health activists
CFIR: Consolidated Framework for Implementation Research
DF/DHF: Dengue fever/dengue haemorrhagic fever
EPPI-Community engagement: Evidence for Policy Practice Information and Coordinating Community engagement
FGD: Focus group discussion
IDIs: In-depth interviews
LMIC: Low- and middle-income countries
PRISMA: Preferred Reporting Items for Systematic Review
ROBINS -I: Risk Of Bias In Non-randomised Studies-of Interventions
RTA: Reflexive thematic analysis
SEA: South-East Asia
TDR: Special Programme for Research and Training in Tropical Diseases

## Declarations

Formal consent was waved as the study included only published data, but not any human or animal participants. Hence, formal consent was not necessary to obtain.
