# Supplementary material for: Facilitators and barriers to engaging communities in health service research on dengue control in Indo-Pacific region: a systematic review
Source: BMC Public Health. 2023 Oct 5;23:1924. doi: 10.1186/s12889-023-16845-8 (PMC10552252; doi:10.1186/s12889-023-16845-8)
Supplement: Supplementary file 1 — Supplementary Material 1 [file 12889_2023_16845_MOESM1_ESM.doc]

Supplementary Table

**table s1. Classification of the low-and middle-income countries**

| The World Bank assigns the world’s economies to four income groups—low, lower-middle, upper-middle, and high-income countries. The classifications are updated each year on July 1 and are based on GNI per capita in current USD (using the [Atlas method](https://datahelpdesk.worldbank.org/knowledgebase/articles/77933-what-is-the-world-bank-atlas-method) exchange rates) of the previous year (i.e. 2019 in this case). |
| --- |
| For the current 2024 fiscal year, low-income economies are defined as those with a GNI per capita, calculated using the World Bank Atlas method, of $1,135 or less in 2022; **lower middle-income** economies are those with a GNI per capita between $1,136 and $4,465; **upper middle-income** economies are those with a GNI per capita between $4,466 and $13,845; high-income economies are those with a GNI per capita of $13,846 or more. |

***listing of countries that are defined as the “Indo-Pacific” region***

| Australia |
| --- |
| Bangladesh |
| Bhutan |
| Brunei |
| Cambodia |
| Fiji |
| India |
| Indonesia |
| Japan |
| Laos |
| Malaysia |
| Maldives |
| Myanmar |
| Nepal |
| New Zealand |
| Papua New Guinea, |
| Philippines |
| Singapore |
| Sri Lanka |
| Taiwan |
| Thailand |
| Timor Leste |
| United States |
| Vietnam |

Source: [Indo-Pacific Region - CEOWORLD magazine](https://ceoworld.biz/indo-pacific/).
