# Supplementary material for: Facilitators and barriers to engaging communities in health service research on dengue control in Indo-Pacific region: a systematic review
Source: BMC Public Health. 2023 Oct 5;23:1924. doi: 10.1186/s12889-023-16845-8 (PMC10552252; doi:10.1186/s12889-023-16845-8)
Supplement: Supplementary file 3 — Supplementary Material 3 [file 12889_2023_16845_MOESM3_ESM.doc]

Supplementary Table

**table s2. Search strategy in PubMed**

| Search number | Query |
| --- | --- |
| 14 | (#11) AND (#12) |
| 13 | degnue - Spellcheck off |
| 12 | dengue |
| 11 | (((((((((#1) OR (#2)) OR (#3)) OR (#4)) OR (#5)) OR (#6)) OR (#7)) OR (#8)) OR (#9)) OR (#10) |
| 10 | cooperative inquiry |
| 9 | action inquiry |
| 8 | action science |
| 7 | community-based research |
| 6 | participatory action research |
| 5 | participatory research |
| 4 | action research |
| 3 | participatory |
| 2 | community engagement |
| 1 | community |
