# Supplementary material for: Facilitators and barriers to engaging communities in health service research on dengue control in Indo-Pacific region: a systematic review
Source: BMC Public Health. 2023 Oct 5;23:1924. doi: 10.1186/s12889-023-16845-8 (PMC10552252; doi:10.1186/s12889-023-16845-8)
Supplement: Supplementary file 4 — Supplementary Material 4 [file 12889_2023_16845_MOESM4_ESM.doc]

**Supplementary information**

**table s3. Excluded studies**

| Sr.No | Study, year | Main reason for exclusion | References |
| --- | --- | --- | --- |
| 1 | Swaddiwudhipong, 2012 | assessment of HE programe | Swaddiwudhipong W, Chaovakiratipong C, Nguntra P, et al. Effect of health education on community participation in control of dengue hemorrhagic fever in an urban area of Thailand. Southeast Asian J Trop Med Public Health 1992; 23:200-6. |
| 2 | Dhar-Chowdhury, 2014 | knowledge about dengue transmission | Dhar-Chowdhury P, Emdad Haque C, Michelle Driedger S, et al. Community perspectives on dengue transmission in the city of Dhaka, Bangladesh. Int Health. 2014;6:306-16. |
| 3 | Ahsan,2021 | letter to editors | Atik Ahsan, Najmul Haider, Richard Kock, et al. Possible drivers of the 2019 dengue outbreak in Bangladesh: the need for a robust community-level surveillance system, Journal of Medical Entomology*,* 2021; 58:37–9. |
| 4 | Sulistyawati,2019 | KAP assessment | Sulistyawati S, Dwi Astuti F, Rahmah Umniyati S, et al. Dengue vector control through community empowerment: lessons learned from a community-based study in Yogyakarta, Indonesia. Int J Environ Res Public Health. 2019;16(6):1013. |
| 5 | Sayono,2019 | survey responds only, not CE | Sayono S, Widoyono W, Sumanto D, et al. Impact of dengue surveillance workers on community participation and satisfaction of dengue virus control measures in Semarang municipality, Indonesia: a policy breakthrough in public health action. Osong Public Health Res Perspect. 2019;10(6):376–84. |
| 6 | Paz-Soldan,2016 | ovitrap testing | Paz-Soldan VA, Yukich J, Soonthorndhada A, et al. Design and testing of novel lethal ovitrap to reduce populations of aedes mosquitoes: community-based participatory research between industry, academia and communities in Peru and Thailand. PLoS ONE 2016; 11(8): e0160386. |
| 7 | Griffiths, 2013 | case study | [Karolina Griffiths](https://www.semanticscholar.org/author/Karolina-Griffiths/40377191), [M. Banjara](https://www.semanticscholar.org/author/M.-Banjara/3933407), [T. O'Dempsey](https://www.semanticscholar.org/author/T.-O'Dempsey/1398062988), [B. et al.](https://www.semanticscholar.org/author/B.-Munslow/81047509) [Public Health Responses to a dengue outbreak in a fragile state: a case study of Nepal](https://www.semanticscholar.org/paper/Public-Health-Responses-to-a-Dengue-Outbreak-in-a-A-Griffiths-Banjara/b5337584e4bb9c453587e46a52513c2813a844a8). Journal of Tropical Medicine 2013. Article ID 158462 |
| 8 | Babu,2019 | monitoring disease trends, | Babu AN, Niehaus E, Shah S, et al. Smartphone geospatial apps for dengue control, prevention, prediction, and education: MOSapp, DISapp, and the mosquito perception index (MPI). Environ Monit Assess. 2019;191(Suppl 2):393. |
| 9 | van den Berg, 2012 | stratification, integration of vector control | van den Berg, H., Velayudhan, R., Ebol, A. *et al.* Operational efficiency and sustainability of vector control of malaria and dengue: descriptive case studies from the Philippines. Malar J 2012” 11, 269 |
| 10 | Tana, 2012 | assessment of the innovative process and results. | Tana S, Umniyati S, Petzold M, et al. Building and analyzing an innovative community-centered dengue-ecosystem management intervention in Yogyakarta, Indonesia. Pathog Glob Health. 2012 ;106(8):469-78. |
| 11 | Arunachalam, 2012 | cluster randomized controlled trial for efficacy assessment | Arunachalam N, Tyagi BK, Samuel M, et al. Community-based control of Aedes aegypti by adoption of eco-health methods in Chennai City, India. Pathog Glob Health. 2012;106(8):488-96. |
| 12 | Khun.2007 | process of HE program | Khun S, Manderson L. Community and school-based health education for dengue control in rural Cambodia: a process evaluation. PLoS Negl Trop Dis. 2007;1(3):e143. |
| 13 | Bhattarai,2019 | acceptability of mobile SMS | Bhattarai AH, Sanjaya GY, Khadka A et al. The addition of mobile SMS effectively improves dengue prevention practices in community: an implementation study in Nepal. BMC Health Serv Res 2019: 19, 699 |
| 14 | Brusich,2015 | situation analysis | Brusich M, Grieco J, Penney N, Tisgratog R, et al. Targeting educational campaigns for prevention of malaria and dengue fever: an assessment in Thailand. Parasit Vectors. 2015;8:43. |
| 15 | Phuanukoonnon,  2006 | health belief model constructs | Phuanukoonnon S, Brough M, Bryan JH. Folk knowledge about dengue mosquitoes and contributions of health belief model in dengue control promotion in Northeast Thailand. Acta Trop. 2006;99(1):6-14. |
| 16 | Mahilum, 2005 | program evaluation | Mahilum MM, Ludwig M, Madon MB, et al. Evaluation of the present dengue situation and control strategies against Aedes aegypti in Cebu City, Philippines. J Vector Ecol. 2005;30(2):277-83. |
| 17 | Boonchutima,2017 | trend analysis | Boonchutima S, Kachentawa K, Limpavithayakul M, et al. Longitudinal study of Thai people media exposure, knowledge, and behavior on dengue fever prevention and control. J Infect Public Health. 2017;10(6):836-41. |
| 18 | Sim, 2020 | environmental aspect | Sim S, Ng LC, Lindsay SW, et al. A greener vision for vector control: The example of the Singapore dengue control programme. Plos Negl Trop Dis. 2020 ;14(8):e0008428. |
| 19 | Zinszer,2020 | outside the region of our focus | Zinszer K, Caprara A, Lima A, et al Sustainable, healthy cities: protocol of a mixed methods evaluation of a cluster randomized controlled trial for Aedes control in Brazil using a community mobilization approach. Trials. 2020;21(1):182. |
| 20 | Suwanbamrung,2020 | assessment of the guideline usage | Suwanbamrung C, Le CN, Maneerattanasak S, et al. Developing and using a dengue patient care guideline for patients admitted from households to primary care units and the district hospital: A community participatory approach in Southern Thailand. One Health. 2020 17; 10:100168. |
| 21 | Manaf, 2021 | elites only, not covered general community | Manaf RA., Mahmud A, NTR A. et al. A qualitative study of governance predicament on dengue prevention and control in Malaysia: the elite experience. BMC Public Health 2021: 21, 876 |
